# Investigations of an inducible intact dystrophin gene excision system in cardiac and skeletal muscle in vivo

**DOI:** 10.1038/s41598-020-67372-0

**Published:** 2020-07-03

**Authors:** Addeli Bez Batti Angulski, John Bauer, Houda Cohen, Kazuhiro Kobuke, Kevin P. Campbell, Joseph M. Metzger

**Affiliations:** 1 Department of Integrative Biology and Physiology, University of Minnesota Medical School, 6-125 Jackson Hall, 321 Church Street SE, Minneapolis, MN 55455 USA; 2 Howard Hughes Medical Institute, Senator Paul D. Wellstone Muscular Dystrophy Specialized Research Center, Department of Molecular Physiology and Biophysics and Department of Neurology, Roy J. and Lucille A. Carver College of Medicine, The University of Iowa, Iowa City, IA 52242 USA

**Keywords:** Biological techniques, Physiology

## Abstract

We sought here to induce the excision of a large intragenic segment within the intact dystrophin gene locus, with the ultimate goal to elucidate dystrophin protein function and stability in striated muscles in vivo. To this end, we implemented an inducible-gene excision methodology using a floxed allele approach, demarcated by dystrophin exons 2–79, in complementation with a cardiac and skeletal muscle directed gene deletion system for spatial–temporal control of dystrophin gene excision in vivo. Main findings of this study include evidence of significant intact dystrophin gene excision, ranging from ~ 25% in heart muscle to ~ 30–35% in skeletal muscles in vivo. Results show that despite evidence of significant dystrophin gene excision, no significant decrease in dystrophin protein content was evident by Western blot analysis, at three months post excision in skeletal muscles or by 6 months post gene excision in heart muscle. Challenges of in vivo dystrophin gene excision revealed acute deleterious effects of tamoxifen on striated muscles, including a transient down regulation in dystrophin gene transcription in the absence of dystrophin gene excision. In addition, technical limitations of incomplete dystrophin gene excision became apparent that, in turn, tempered interpretation. Collectively, these findings are in keeping with earlier studies suggesting the dystrophin protein to be long-lived in striated muscles in vivo; however, more rigorous quantitative analysis of dystrophin stability in vivo will require future works in which more complete gene excision can be demonstrated, and without significant off-target effects of the gene deletion experimental platform per se.

## Introduction

Duchene muscular dystrophy (DMD) is an X-linked inherited disease characterized by a fragile muscle membrane and progressive striated muscle deterioration, affecting ~ 1:5,000 newborn males worldwide^1,2^. DMD boys have severe skeletal muscle wasting early in life and loss of ambulation and wheelchair dependency occur by the teenage years. DMD is fatal by the third decade of life due to cardio-respiratory failure^2^. DMD is caused by mutations in the dystrophin gene, which encodes a large multi-domain cytoskeletal protein that functions intracellularly in muscle to stabilize the muscle membrane against the mechanical forces associated with muscle stretch and contraction^3,4^. The *DMD* gene is the largest known human gene, spanning ~ 2.5 megabases of DNA. The dystrophin gene encompasses 79 exons that are spliced together to give rise to a 427 kDa protein containing 3,685 amino acids.

Numerous experimental therapeutic strategies have been undertaken in attempt to rescue dystrophin deficiency in animal models of DMD, including premature stop codon suppression, exon skipping to restore a functional reading frame and gene addition therapy^5–7^. One of the most promising approaches has been the expression of truncated dystrophin genes through adeno-associated viral (AAV) delivery^8,9^. Shortened dystrophin transgenes, coding partially functional micro-dystrophins contain essential domains of the dystrophin protein have been generated to be compatible with the limited carrying capacity of rAAV vectors^10^. Because gene replacement therapies are subject to clearance and turnover of dystrophin protein and muscle fibers, it is likely that repeated, lifelong injections will be required to maintain therapeutic effects. Therefore, insight into the turnover and stability of dystrophin in vivo is essential to determine duration of action effects of dystrophin as this would be important in guiding therapeutic dosing regimens in DMD patients. While studies on whole-animal and clinical studies targeting gene replacement therapy have led to significant progress in unravelling the efficiency and functionality of truncated dystrophins, the in vivo half-life of dystrophin remains poorly understood.

An often cited study by Ahmad and co-workers showed evidence of dystrophin protein detection and localization to the sarcolemmal membrane at 26 weeks after somatic termination of dystrophin gene transcription. This is taken as evidence that dystrophin has a long half-life in skeletal muscle tissues in vivo^11^. Seno and colleagues demonstrated that several months were required to reduce dystrophin protein expression after a specific dystrophin knockdown using adeno-associated virus (AAV)-mediated RNA interference (RNAi)^12^. Together, these studies suggest that full length dystrophin appears highly stable, indicating an apparent long half-life of dystrophin in vivo.

Whereas these previous works provide qualitative estimates of intact dystrophin stability in vivo, there have yet to be study designs for quantitative assessment of dystrophin stability in skeletal and cardiac muscles of live animals. It is presently unknown and critical to determine, whether quantitative methodologies could be developed to precisely determine dystrophin half-life in vivo.

To begin to address this important question we have implemented a methodology using a floxed allele approach, together with a cardiac or skeletal muscle directed MerCreMer transgene expression to accomplish spatial–temporal control of full-length dystrophin gene excision in vivo. In this study we sought to induce the excision of a large intragenic locus within the dystrophin gene in the heart and skeletal muscle tissues in order to be able to investigate dystrophin half-life in vivo. We found that even with significant refinement in the gene excision procedure the largest amount of full-length dystrophin gene excision achieved was 25–35%. Interestingly, this level of intact dystrophin gene excision was insufficient to cause any detectable reduction in dystrophin protein content in striated muscles, up to 6 months post gene excision in the heart. In addition, challenges of in vivo dystrophin locus excision revealed the deleterious acute effects of large doses of tamoxifen on striated muscles, including a transient down regulation in dystrophin gene transcription in the absence of dystrophin gene excision.

## Results

### Generation of mice

**Figure 1:**
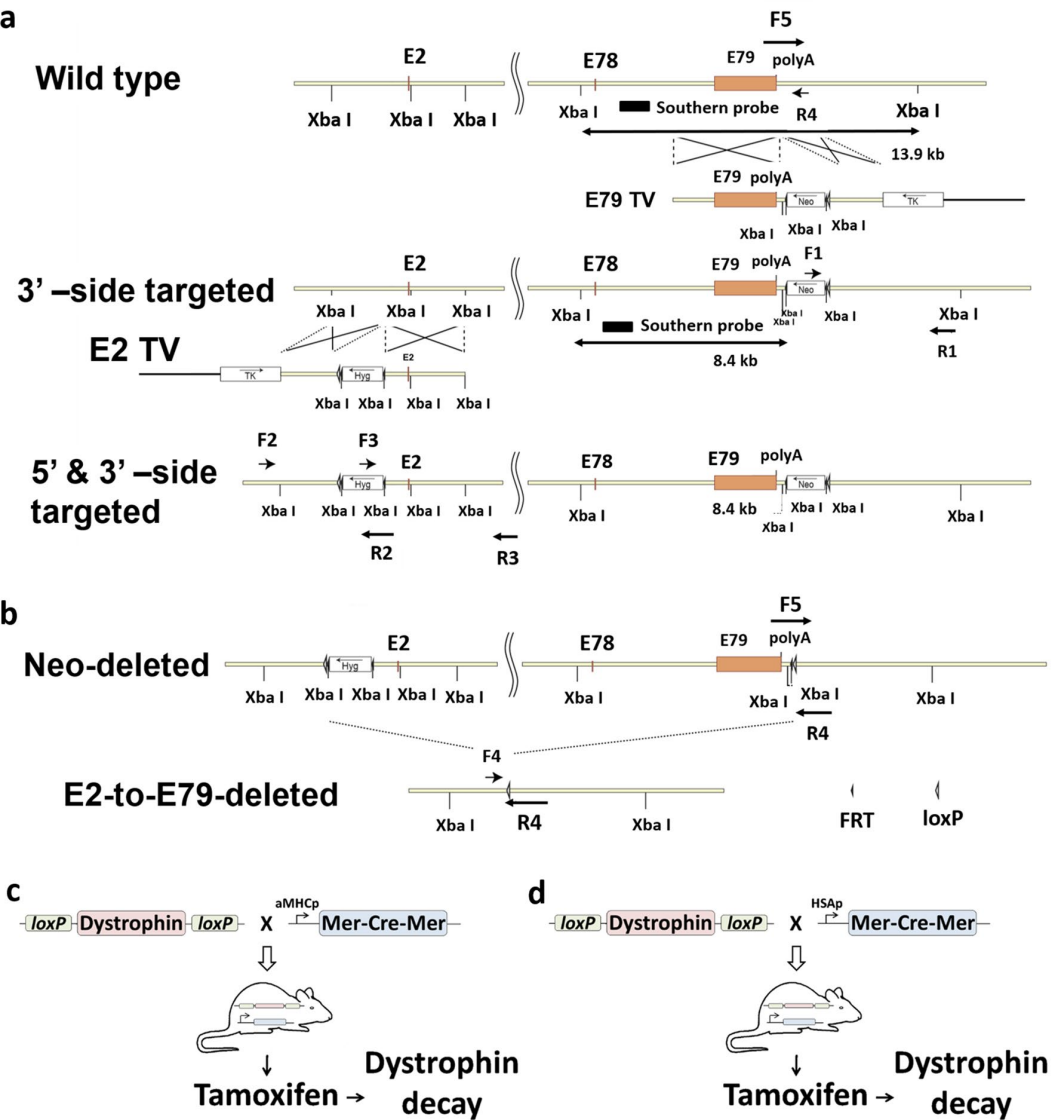
Strategy for the generation of floxed dystrophin mice and establishment of inducible Cre recombinant mice. (**a**) Schematic presentation of the steps used to generate conditional knockout mice. The schematic drawing shows the unmodified wild type dystrophin gene. Targeting of the genomic locus by homologous recombination generates a modified gene that carries the correctly placed loxP sites (indicated by white arrows). A hygromycin (Hyg) or a neomycin (Neo) resistance gene is linked to the first or second loxP site, respectively, for positive selection during gene targeting. Primers detailed in "Materials and methods" section. (**b**) Neomycin resistance cassette was excised. (**c**) Floxed dystrophin mice (floxed Dys) were crossed with mice expressing a cardiac-specific tamoxifen-inducible Cre recombinase (αMHC.MerCreMer) to achieve cardiac specific gene excision. (**d**) Floxed dystrophin mice were crossed with mice expressing a skeletal muscle-specific tamoxifen-inducible Cre recombinase (HSA.MerCreMer) to accomplish skeletal muscle specific gene excision. Tamoxifen was administered to the resulting floxed Dys × MCM mice to investigate dystrophin gene excision efficiency. F1–F5 and R1–R4, PCR primers described in "Materials and methods" section.

In order to enable conditional knockout of the whole dystrophin locus, two loxP sites were serially inserted into the dystrophin locus by homologous recombination. The details are described in "Materials and methods" section; briefly a loxP and a flrted neomycin cassette were introduced into the 3′-side of exon 79, then another loxP and a flrted hygromycin cassette into the 5′-side of exon 2 in ES cells. Transfection of FLPe expression vector successfully deleted the flrted neomycin cassette, but extensive efforts so far have failed to remove the flrted hygromycin cassette without disturbing potency of germiline-transmission of ES cells. (Fig. 1a,b). The dystrophin-floxed ES cell clone was microinjected into C57BL/6J blastocysts, and chimeric mice were obtained. Only hemizygous males and homozygous females under C57BL/6J background were used for further experiments.

To investigate dystrophin gene excision efficiency in heart and skeletal muscle, floxed dystrophin mice were crossed with mice expressing a cardiac or skeletal muscle-specific tamoxifen-inducible Cre recombinase transgene (MerCreMer) (Fig. 1c and d). In this experimental model, induction of a spatial–temporal excision of dystrophin gene was made via tamoxifen administration.

### In vivo efficiency of dystrophin gene excision

**Figure 2:**
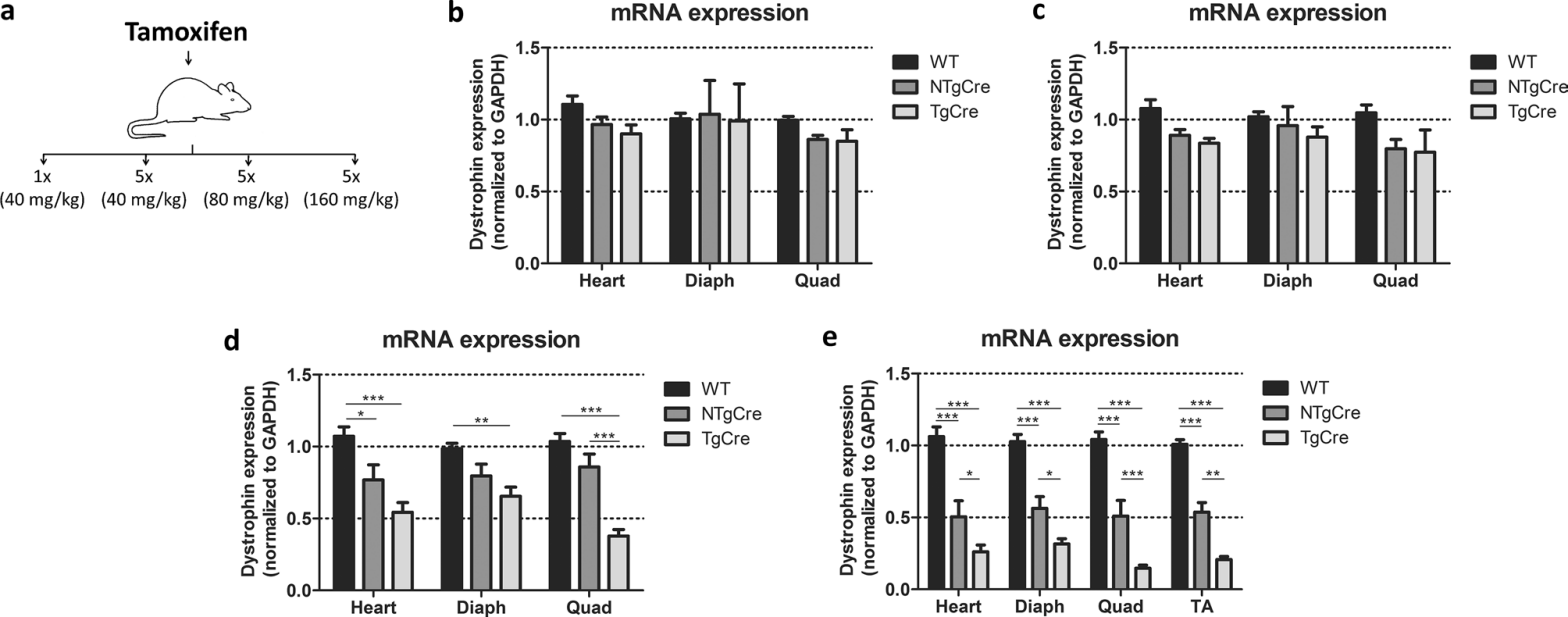
Dystrophin mRNA expression in skeletal/cardiac muscle at 5 day point, post tamoxifen injection. (**a**) Schematic representation of different tamoxifen injection protocols, varying from 1 single injection to five consecutive injections that were used to determine the most efficient protocol to induce dystrophin gene excision in vivo. (**b**) Dystrophin mRNA expression after 1 single injection of tamoxifen at 40 mg/kg. (**c**)–(**e**) Dystrophin mRNA expression after 5 consecutive days of tamoxifen at 40 mg/kg, 80 mg/kg and 160 mg/kg, respectively. Per injection protocol three to five mice were analyzed and ΔCT-values were normalized to the mean value of WT (C57BL/6J) mice. Dystrophin mRNA expression normalized to GAPDH. The error bar correlates to the SEM of the biological replicates (*P < 0.05; **P < 0.01; ***P < 0.001). WT: non-treated wild type (C57BL/6J), NTgCre: tamoxifen-treated floxed dystrophin without Cre transgene, TgCre: tamoxifen-treated floxed dystrophin with Cre transgene.

To estimate the in vivo efficacy of tamoxifen to induce dystrophin gene excision, several different tamoxifen injection protocols were tested, varying from one single injection at 40 mg/kg to five consecutive injections at 160 mg/kg (Fig. 2a). To determine the gene excision efficiency, we performed quantitative RT-PCR to investigate the reduction of dystrophin expression on the transcriptional level. Thus, mRNA levels were used as a surrogate measurement of dystrophin gene locus inactivation, similar to past studies excising DGC genes^13^. One single dose at 40 mg/kg did not show a significant reduction of the mRNA expression levels in the heart and skeletal muscle tissues (Fig. 2b). Consecutive injections of TAM are frequently used to achieve higher recombination efficiencies. Therefore, we injected tamoxifen for five consecutive days at different concentrations and determined the mRNA levels after 5 days of treatment. Induction of the nuclear translocation of Cre recombinase through tamoxifen administration at 40 mg/kg on 5 consecutive days resulted in a poor efficiency excision of the dystrophin gene in heart and skeletal muscle tissues (Fig. 2c). After five tamoxifen injections at 80 mg/kg mRNA expression was significantly reduced in heart and skeletal muscle tissues of transgenic Cre mice (TgCre: floxed dystrophin with Cre transgene) (Fig. 2d). Increasing the dose of tamoxifen from 80 mg/kg to 160 mg/kg improved dystrophin gene excision efficiency, especially in skeletal muscle tissues, but did not result in 100% of excision (Fig. 2e).

It has been described that tamoxifen can have significant off-target effects, including effects on gene transcription^14,15^. We therefore, included a non-treated wild type C57BL/6J (WT) group in our experiments and compared dystrophin mRNA expression between tamoxifen-treated non-transgenic Cre (NTgCre: floxed dystrophin without Cre transgene) and TgCre mice with non-treated WT mice to test whether tamoxifen would affect gene transcription by using dystrophin mRNA abundance as a parameter. Surprisingly, we found major differences in mRNA expression between tamoxifen-treated NTgCre samples, as compared to non-treated WT mice at a dose of 160 mg/kg. The RT-qPCR data showed that dystrophin mRNA levels of tamoxifen-treated NTgCre were transiently downregulated to 55%, 46%, 47% and 53% in heart, diaphragm, TA and quadriceps, respectively, compared with non-treated WT mice (*P* < 0.001, Fig. 2e), demonstrating evidence that dystrophin mRNA expression is being affected by tamoxifen at 160 mg/kg and that effect is independent of Cre recombinase transgene. This has, to our knowledge, not been reported previously in the context of tamoxifen regulating dystrophin transcription and raises important questions regarding the choice of controls to tamoxifen-treated experiments. Because we observed an isolated effect of tamoxifen in regulating dystrophin mRNA levels, we next compared dystrophin mRNA expression between tamoxifen-treated NTgCre and TgCre mice in order to evaluate authentic dystrophin gene excision 5 days after last tamoxifen injection. Here RT-qPCR analysis showed that mRNA levels were significantly reduced in TgCre mice compared with NTgCre, with gene excision efficiency varying from 25% in heart and diaphragm (*P* < 0.05) to 33% in TA (*P* < 0.01) and 36% in quadriceps (*P* < 0.001) (Fig. 2e). Collectively, these data show that increasing the number of injections and tamoxifen dose resulted in significant dystrophin gene excision, but in no experiments was it found that 100% of dystrophin gene was excised and emphasizes the importance of appropriate control groups in tamoxifen-treated experiments, especially when high doses of tamoxifen are being used.

### Evaluation of long-term effects of tamoxifen on gene transcription

**Figure 3:**
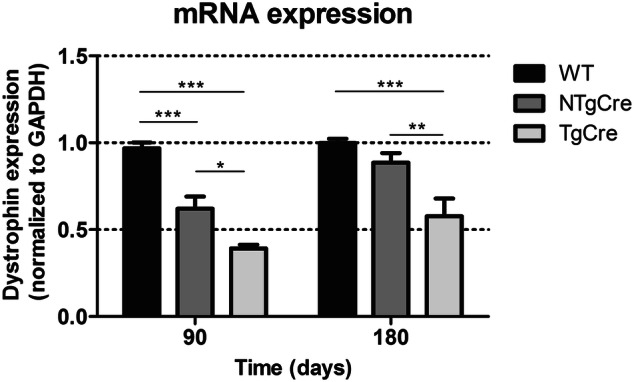
Dystrophin mRNA expression in cardiac tissue. Dystrophin mRNA expression in the heart at 90 and 180 days after last day of tamoxifen injection (160 mg/kg on five consecutive days)**.** Five to seven mice were analyzed and ΔCT-values were normalized to the mean value of WT (C57BL/6J) mice. Dystrophin mRNA expression normalized to GAPDH. The error bar correlates to the SEM of the biological replicates (*P < 0.05; **P < 0.01; ***P < 0.001). WT: non-treated wild type (C57BL/6J), NTgCre: tamoxifen-treated floxed dystrophin without Cre transgene, TgCre: tamoxifen-treated floxed dystrophin with Cre transgene.

To evaluate the possible longer-term effects of tamoxifen in regulating dystrophin gene transcription (independently of dystrophin gene excision), dystrophin mRNA content was investigated by RT-qPCR 90 and 180 days after the last tamoxifen injection. Because we observed a tamoxifen-dependent effect on dystrophin gene transcription 5 days after last injection (Fig. 2e NTgCre data), we further investigated whether tamoxifen at 160 mg/kg is having a prolonged effect in regulating dystrophin mRNA over time. Unexpectedly, the RT-qPCR data demonstrated that at 90 days after last tamoxifen treatment mRNA level was significantly decreased by 33% in tamoxifen-treated NTgCre hearts compared with WT (*P* < 0.001, Fig. 3). At 180 day, however, the dystrophin mRNA expression of tamoxifen-treated NTgCre hearts was not significantly decreased compared to WT (Fig. 3).

Whereas we have observed a tamoxifen effect on dystrophin gene transcription, it is worth mentioning that calculations of intact dystrophin gene excision did not differ between 5 (25%) (Fig. 2e), 90 (23%) and 180 (30%) days after last tamoxifen injection (Fig. 3), a result consistent with the post-mitotic status of the adult myocardium. Owing to the marked effect of TAM to reduce dystrophin mRNA, estimating dystrophin gene excision efficiency by tracking the total pool of dystrophin mRNA was made more compelx. As the NTgCre dystrophin mRNA group reflects the the TAM-based effect and the TgCre group represents the TAM effect + the dystrophin gene excision effect, subtracting the TgCre value from the NTgCre value gives the estimated dystrophin gene excision value, as reported here.

**Figure 4:**
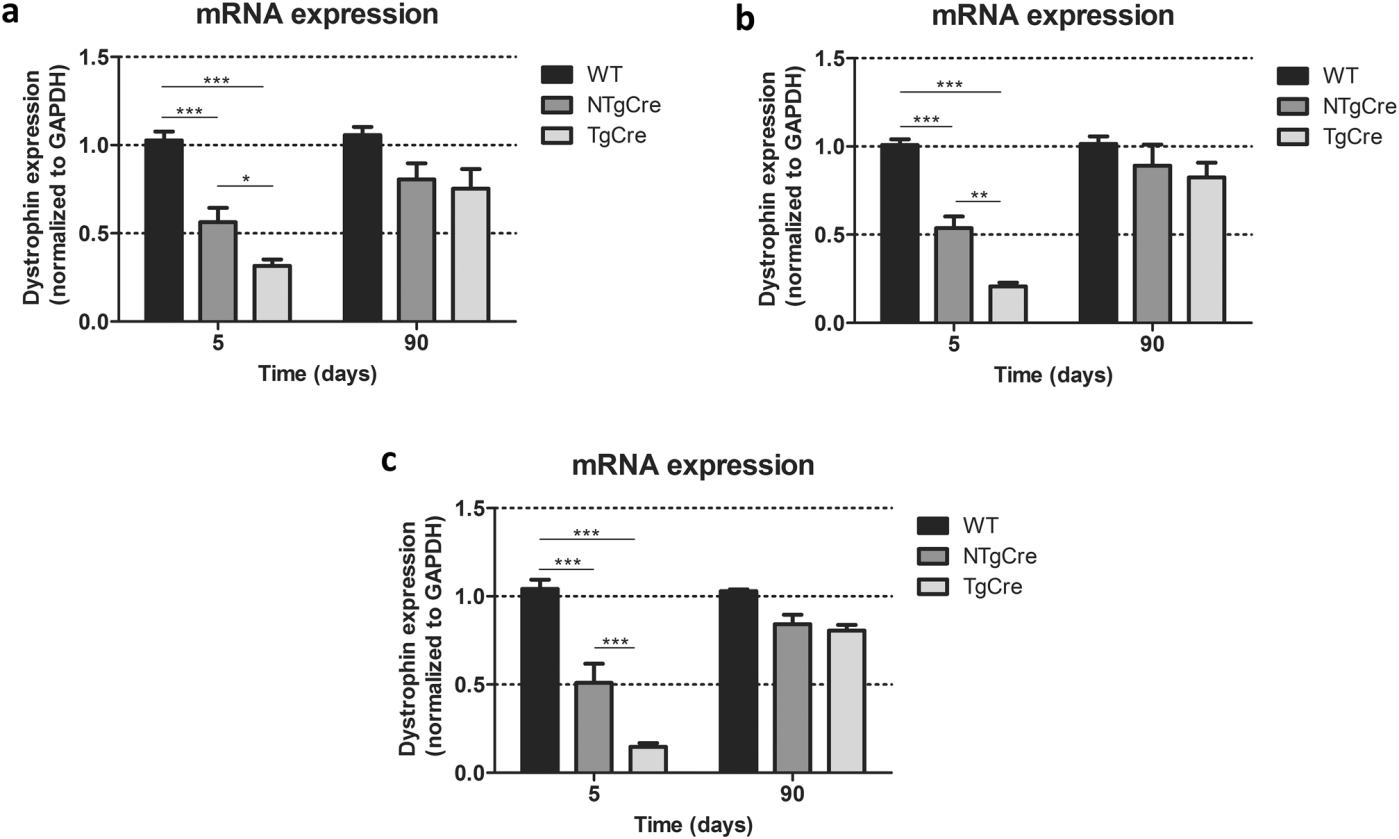
Dystrophin mRNA expression in skeletal muscle tissues. (**a**) Dystrophin mRNA expression in the diaphragm 5 and 90 days after last day of tamoxifen injection (160 mg/kg on five consecutive days). (**b**) Dystrophin mRNA expression in the TA 5 and 90 days after last day of tamoxifen injection. (**c**) Dystrophin mRNA expression in the quadriceps 5 and 90 days after last day of tamoxifen injection. Five to seven mice were analyzed and ΔCT-values were normalized to the mean value of WT (C57BL/6J) mice. Dystrophin mRNA expression normalized to GAPDH. The error bar correlates to the SEM of the biological replicates (*P < 0.05; **P < 0.01; ***P < 0.001). WT: non-treated wild type (C57BL/6J), NTgCre: tamoxifen-treated floxed dystrophin without Cre transgene, TgCre: tamoxifen-treated floxed dystrophin with Cre transgene.

In contrast to the heart, dystrophin mRNA expression was not significantly reduced in diaphragm, TA and quadriceps at 90 days after last tamoxifen injection (Fig. 4a–c). Furthermore, the RT-qPCR analysis of skeletal muscle tissues demonstrated that the dystrophin mRNA levels in the tamoxifen-treated TgCre group were restored back to controls (WT and NTgCre) at 90 days after last tamoxifen injection, suggesting that repopulation of myofibers did occur over time. To this end, we decided not to perform RT-qPCR and Western blot analysis 180 days after last tamoxifen treatment as we did for the heart. Together, these results indicate a transient tamoxifen-dependent effect on dystrophin gene transcription in skeletal muscle, demonstrating that dystrophin mRNA levels are restored by 90 days post-injection.

In addition, to assess for any potential histologic changes due to tamoxifen treatment and gene excision we performed Hematoxylin and Eosin (H&E) staining. H&E staining revealed normal histology in tissue sections from heart and quadriceps after tamoxifen treatment and gene excision (Supplemental Figure S5). No remarkable changes were observed in heart and quadriceps of non-transgenic Cre and transgenic Cre-treated mice (90 and 180 days).

### Dystrophin protein content post-gene excision in heart and skeletal muscles in vivo

We observed a tamoxifen-dependent effect in down-regulating dystrophin gene transcription at 160 mg/kg, among all the protocols that we tested, and this was the only protocol tested demonstrating statistically significant dystrophin gene excision in vivo. It has been suggested that intact dystrophin is highly stable once it reaches the sarcolemma^11,12^, but the in vivo tissue half-life of full length dystrophin has not been accurately determined and still remains poorly understood. Because one of the main goals of this study was to investigate in vivo dystrophin half-life, we investigated full-length dystrophin protein expression in the heart and skeletal muscle tissues after 90 and 180 days (heart only) of gene excision induction by tamoxifen injections at 160 mg/kg on 5 consecutive days.

**Figure 5:**
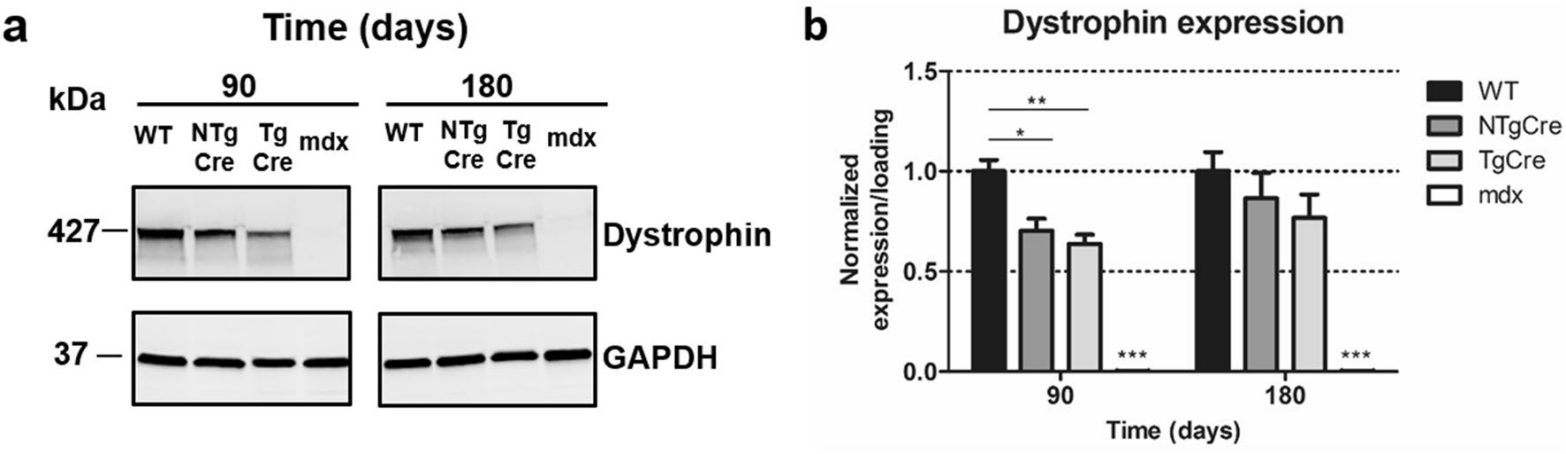
Dystrophin protein excision in the heart. (**a**) Representative Western blot of total protein from heart samples 90 and 180 days after last tamoxifen injection (160 mg/kg on five consecutive days). Blots were probed with antibodies to dystrophin (mid rod) and GAPDH as loading controls. The image presented in this figure represent the cropped gel. Full-length blots/gels are presented in Supplementary Figure S1. (**b**) Densitometric quantification of blots in A. Expression of dystrophin was quantified relative to loading and normalized to WT. Values represent mean ± standard error of the mean of dystrophin. *n* = 5–7 (*P < 0.05; **P < 0.01; ***P < 0.001). WT: non-treated wild type (C57BL/6J), NTgCre: tamoxifen-treated floxed dystrophin without Cre transgene, TgCre: tamoxifen-treated floxed dystrophin with Cre transgene, mdx: Duchene muscular dystrophin mouse model.

In cardiac tissues at 90 days post tamoxifen, Western blot analysis showed a statistically significant difference between tamoxifen-treated and WT mice, but no significant difference was observed between NTgCre and TgCre (Fig. 5a,b). This significant difference between tamoxifen-treated NTgCre and WT mice provides evidence that dystrophin protein content is being affected by tamoxifen at 160 mg/kg and that effect is independent of Cre recombinase transgene, consistent with the RT-qPCR results (Fig. 3). At 180 days, however, dystrophin protein expression of tamoxifen-treated hearts was slightly lower, but not significantly decreased compared to WT (Fig. 5a,–b).

**Figure 6:**
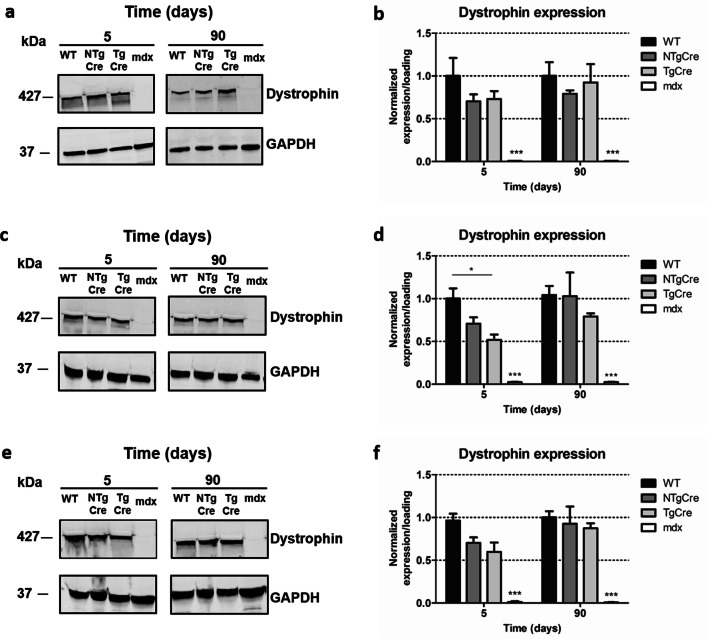
Dystrophin protein excision in skeletal muscle tissues. Representative Western blot of total protein from diaphragm (**a**), tibialis anterior (**c**) and quadriceps (**e**) 5 and 90 days after last tamoxifen injection (160 mg/kg on five consecutive days). Blots were probed with antibodies to dystrophin (mid rod) and GAPDH as loading controls. The images presented in this figure represent the cropped gels. Full-length blots/gels are presented in Supplementary Figures S2, S3 and S4. (**b**) Densitometric quantification of blots in A. (**d**) Densitometric quantification of blots in C. (**f**) Densitometric quantification of blots in E. Expression of dystrophin was quantified relative to loading and normalized to WT. Values represent mean ± standard error of the mean of dystrophin. *n* = 5–7 (*P < 0.05; **P < 0.01; ***P < 0.001). WT: non-treated wild type (C57BL/6J), NTgCre: tamoxifen-treated floxed dystrophin without Cre transgene, TgCre: tamoxifen-treated floxed dystrophin with Cre transgene, mdx: Duchene muscular dystrophin mouse model.

In contrast to the heart, dystrophin protein expression of NTgCre came back up to WT in all skeletal muscle tissues evaluated 90 days after last tamoxifen injection (Fig. 6). Furthermore, no significant difference was observed between NTgCre and TgCre 90 days post treatment. Together, these data show that despite evidence of significant dystrophin gene excision, no significant decrease in dystrophin protein content was evident, at 90 days post excision in skeletal muscles or by 180 days post gene excision in the heart. Furthermore, our results demonstrate evidence of a tamoxifen-dependent effect on dystrophin protein content in the heart at 90 days post tamoxifen treatment but also shows that tamoxifen’s effect on dystrophin mRNA was no longer evident 90 and 180 days.

### Tamoxifen effects on cardiac function

Tamoxifen-induced cardiomyopathy in Tg (MerCreMer) mice has been proposed to be Mer-dependent by causing an increase in nuclear tamoxifen accumulation. In vivo hemodynamics data also suggest an independent effect of tamoxifen to suppress cardiac function^16^. We therefore determine whether our tamoxifen injection protocol at a dose of 160 mg/kg on five consecutive days resulted in cardiac toxicity in floxed Dys x αMHC.MerCreMer (NTg and Tg) and floxed Dys x HSA.MerCreMer (NTg and Tg) mice. We assessed myocardial function by echocardiography in WT mice in the absence of tamoxifen and in tamoxifen-treated mice at 5, 90 and 180 after last tamoxifen injection.

Echocardiography analysis of floxed Dys × αMHC.MerCreMer mice showed a significant reduction of the LV ejection fraction in NTgCre and in TgCre compared to WT mice 5 days post tamoxifen treatment (Supplementary Fig. S6a). Similarly, LV ejection fraction of floxed Dys × HSA.MerCreMer mice was significantly decrease in NTgCre and in TgCre compared to WT mice (Supplementary Fig. S6b). One prior study reported that tamoxifen induction of the αMHC.MerCreMer strain induces transient myocardial dysfunction, which was resolved after 2 weeks^16^. To determine whether the myocardial dysfunction that we observed in mice 5 days post treatment was transient, we performed echocardiography at 90 and 180 days (αMHC.MerCreMer only). No significant differences were observed between tamoxifen-treated and WT mice at 90 and 180 days post tamoxifen treatment. Collectively, these results suggest that tamoxifen, at a dose of 160 mg/kg on five consecutive days, had a transient effect on cardiac function, with no lasting effect of tamoxifen on suppressing cardiac function at three or 6 months post-treatment.

## Discussion

We sought here to induce the excision of a large intragenic segment within the intact dystrophin gene locus, with the ultimate goal to elucidate dystrophin protein function and stability in striated muscles in vivo. To this end, we implemented an inducible-gene excision methodology using a floxed allele approach, demarcated by dystrophin exons 2–79, together with a cardiac and skeletal muscle directed gene deletion system for spatial–temporal control of dystrophin gene excision in vivo. Main findings include estimates of significant intact dystrophin gene excision, ranging from ~ 25% in heart muscle to ~ 30–35% gene excision in skeletal muscles in vivo. We tracked dystrophin mRNA levels as a surrogate measurement of dystrophin gene excision, similar in strategy to other reports targeting DGC gene excision^13^. To our knowledge, this ~ 2 Mb floxed allele is the largest intragenic DNA sequence deleted in striated muscles in vivo. Results show that despite evidence of significant dystrophin gene excision, as estimated by reduction in dystrophin mRNA levels, no significant decrease in dystrophin protein content was evident by Western blot analysis, at three months post excision in skeletal muscles or by 6 months post gene excision in heart muscle. These findings appear consistent with earlier works suggesting that the full-length dystrophin protein is highly stable in vivo. This point, together with issues related to incomplete intact dystrophin gene excision, along with evidence of off-target effects of tamoxifen, are discussed in below.

Whereas the dystrophin gene-excision protocol developed here did result in evidence of significant dystrophin gene deletion in heart and skeletal muscles in vivo, no significant quantitative changes in dystrophin protein content were evident, even up to 6 months. Upon review of the results, two main experimental challenges became apparent to significantly impact the outcomes of this study. First, despite substantial protocol refinement in terms of modification in tamoxifen dosing and duration regimens, it was not possible to achieve more complete dystrophin gene excision than ~ 25–36% excision, as evaluated by dystrophin mRNA content. This result is in marked contrast to previous published works from our group demonstrating 100% gene excision efficiency for a truncated dystrophin construct in vivo^17^ and for dystroglycan in vivo^13^. Incomplete dystrophin gene excision is particularly complicating in the interpretation of skeletal muscle findings. Here, owing to endogenous skeletal muscle repair/replacement mechanisms in mdx mice, the new muscle fibers are formed with the dystrophin locus intact, which dilute, over time, the pool of myofibers with dystrophin locus excised. Furthermore, incomplete dystrophin gene excision in multinucleated skeletal fibers increases the potential for mosaicism with dystrophin intact/ dystrophin excision nuclei in the same muscle fiber. These outcomes could account for the observation of dystrophin gene excision decreasing over time in skeletal muscle, such that at the 90-day time point, dystrophin mRNA was no longer reduced as compared to controls. It is worth mentioning that incomplete gene excision, as in our study, does have limitations, including the possibility that muscle cells, with dystrophin locus intact, neighboring cells with dystrophin locus excised, could upregulate dystrophin mRNA in a compensatory manner. In this case, we could be underrepresenting our estimates of dystrophin gene excision by tracking mRNA. Genomic DNA analysis is also highly complicated based on the sheer magnitude of the non-muscle cell population in heart and skeletal muscles. Because we used validated skeletal muscle and heart muscle specific promoters directing inducible Cre to only these tissues, then only heart/skeletal myonuclei could have excision and not in non-muscle nuclei. Several papers have shown that cardiac myocytes representing on a per cell basis only 10–30% of the total cell population^18,19^. This, plus knowing that mouse cardiac myocytes are mono- bi-nucleated means cardiac nuclear DNA content is well less than fibroblast/EC/mesenchymal and immune cell DNA content. In skeletal muscle a similar problem arises but is also further complicated by satellite cells in regenerated skeletal muscle known to be dynamic in mdx mice. Collectively, analysis of genomic DNA for quantifying directly the amount of dystrophin gene excision is wrought by these factors that would in turn cloud any meaningful interpretation. A second mitigating factor limiting interpretative insights is evident in the direct effect of tamoxifen to transiently suppress dystrophin mRNA in the absence of dystrophin gene excision.

With these limitations in mind, results from heart muscle may be most instructive in that at 6 months post gene excision, a time point where control data showed no lasting effect of tamoxifen per se on suppressing dystrophin mRNA, a significant gene excision-mediated effect to decrease dystrophin mRNA was still observed. Additionally, the post-mitotic heart muscle, which unlike skeletal muscle does not exhibit significant myocyte turnover, simplifies the interpretation in which we report significant persistent dystrophin gene deletion at the half-year time point. Nonetheless, even within the context of the heart tissue results, a clear analysis of dystrophin protein stability is not forthcoming because of the incomplete dystrophin excision results. Thus, for the heart data, it is not clear whether the unchanged dystrophin content observed is due to (1) long-lived dystrophin protein stability, (2) is evidence of up-regulated dystrophin gene expression, or (3) is some combination of both factors. Ultimately, uncoupling these possibilities will require new experimental models and approaches in which complete or nearly complete dystrophin gene deletion is obtained without mitigating off-target effects of the deletion methodology, similar to what we have demonstrated with efficient excision of modified dystrophin constructs in vivo^17^.

In an attempt to optimize the in vivo efficacy of tamoxifen-mediated dystrophin gene excision, numerous protocols were tested, varying from a single injection at 40 mg/kg to five consecutive injections at high dosing (160 mg/kg). When using a maximal tested dose of tamoxifen on 5 consecutive days at 160 mg/kg we were able to achieve a statistically significant dystrophin gene excision in heart and skeletal muscle tissues. However, increasing the number of injections and tamoxifen concentration did not result in 100% gene excision, with dystrophin excision efficiency varying from 25% in heart and diaphragm to 33% in TA and 36% in quadriceps. The efficiency of Cre/loxP based gene excision has been widely investigated. Overall, two main factors have been shown to affect excision efficiency. First, the nucleotide sequence identity in the spacer region of loxP site that can result in variations of recombination efficiency^20^. The other main factor is the length of DNA between the loxP sites. Several studies have shown that increasing the length of DNA to be excised leads to decreased efficiency of Cre/loxP recombination, likely by regulating the dynamics of the reaction^20,21^. Wang et al.^22^ demonstrated that by varying the length of a DNA fragment flanked by loxP sites from 2 kb up to 13 kb can decrease excision efficiency substantially. The DNA length-dependence of segment deletion has also been observed in the study conducted by Zheng et al., who reported a 11% deletion efficiency for a 4 Mb genetic fragment^21^. The low efficiency observed in the present experiments is most likely due to the ~ 2 Mb intragenic length of DNA that we sought to excise, which reflects that the *DMD* gene is the single largest genetic locus in the human genome spanning ~ 2.5 Mb (0.08% of the human genome). Accordingly, the probability of aligning these two loxP recombinase recognition-sites in a recombination complex is low.

Effects of tamoxifen to alter gene transcription have been described previously^14,15^. Accordingly, as a control, we included in our analyses a non-treated WT (C57BL/6J) group for comparisons of dystrophin mRNA expression between tamoxifen-treated (NTgCre and TgCre) and non-treated WT mice. Using the tamoxifen delivery protocol leading to the most significant amount of dystrophin gene excision, there was a substantial difference in mRNA expression between tamoxifen-treated NTgCre samples compared with non-treated WT mice. Owing to the result of a tamoxifen-dependent effect on dystrophin gene transcription independent of the presence of the Cre transgene suggests that at high dosing tamoxifen has a direct effect to suppress intact dystrophin locus gene expression. Hougen et al.^15^ showed that tamoxifen has a wide-spread effect on cardiac gene transcription and that the effect is greater in response to higher doses. The changes observed in that study, albeit relatively small compared to TAM and MCM in combination, may partially explain the observed effect in regulating dystrophin gene expression showed in this study. The effect of TAM itself in regulating gene transcription has thus far not been fully investigated and will be of interest in future studies to study. As above, this outcome raises a challenge for the investigation of dystrophin half-life in the heart and skeletal muscle tissues in vivo.

Our study did not define the mechanism by which tamoxifen depressed dystrophin expression independently of gene excision. However, the data reported here highlights a significant concern for genomic editing that involves large DNA fragment deletion and raises a challenge for the investigation of dystrophin half-life in the heart and skeletal muscle tissues when large doses of tamoxifen are required. Previously, Dufour et al., demonstrated that in addition to recruiting different nuclear co-activators that can differentially target transcription, tamoxifen can inhibit ERRγ, which along with ERRα, plays a central role in bioenergetic regulation^23^. We speculate that these differences could alter nuclear interactions. It seems clear that the optimal dose and exposure time of tamoxifen to obtain complete gene deletion will vary, depending on the accessibility of the floxed locus, number of copies that has been produced and the level of Cre recombinase expressed. The protocol of DNA recombination should, as also pointed out by Koitabashi et al., be thoroughly investigated in every model with respect to adverse effects and efficacy of target gene inactivation^16^. Ideally, recombination would be possible without non-specific effects on either gene transcription or protein expression.

A final technical consideration relates to the use of tamoxifen as a gene excision regulatory agent. Echocardiographic assessment of tamoxifen treated MerCreMer mice revealed that tamoxifen at a dose of 160 mg/kg on five consecutive days had a transient effect on cardiac function, with no lasting effect of tamoxifen on suppressing cardiac function at 3 or 6 months post treatment. These results are in keeping with the transient dilated cardiomyopathy reported in Tg (αMHC-MerCreMer) mice^16^. In that study, in vivo hemodynamics measurements provide evidence that tamoxifen contributed to the severe cardiac dysfunction, independent of the αMHC-MerCreMer transgene. Here HSA-Cre groups, with or without the Cre transgene, have an equivalent level of reduction in LVEF at the day 5 post TAM treatment. Because HSA directs Cre to skeletal but not the heart muscle, we interpret this as an effect of TAM to reduce LVEF. In comparison, cardiac directed Cre using the same TAM protocol showed slightly more pronounced reduction in LVEF. We interpret this as evidence that in both cardiac and skeletal groups that TAM underlies the LVEF deficits observed. The data obtained in the present study is in agreement with previous work reporting a directed acute effect of tamoxifen on cardiac myocyte contractility and Ca^2+^ handling^24^.

In summary, spatial–temporal in vivo gene excision protocols were developed leading to an estimated excision of 25–35% of the dystrophin gene loci, as estimated by quantifying dystrophin mRNA levels. Targeting the excision of exons 2–79 in the intact dystrophin locus, spanning ~ 2 Mb of DNA, data show significant dystrophin gene excision, ranging from 25 to 36% in skeletal and cardiac muscles in vivo. At up to 6 months post-gene excision, there was no discernable effect on dystrophin protein content, as most evident in the post-mitotic heart study. Whereas this finding is in keeping with earlier studies suggesting dystrophin to be long-lived in striated muscle in vivo, full quantitative accounting of dystrophin stability will await future works in which dystrophin gene excision is more efficient and without complicating effects due to high tamoxifen.

## Materials and methods

### Study design

All animal experiments were performed in accordance with humane practices as called for by the Federal Animal Welfare Act, National Institutes of Health (NIH) guidelines, standards of the Association for Assessment and Accreditation of Laboratory Animal Care (AAALAC International), and at the university level under the regulation of the University Committee on the Use and Care of Animals. They were approved by the University of Minnesota Institutional Animal Care and Use Committee. Statistical analysis and sample size justification was derived from our previous animal studies^24–26^, that were sufficiently powered to obtain significant insights into in vivo studies of muscular dystrophy. In general samples size average 5–7 per group. For qPCR and WB studies, randomization and blinding was carried out by using ear tag or sample ID numbers, which did not indicate genotype. The person carrying out the experiment and subsequent analysis was not aware of the genotypes associated with the ear tag or sample ID until after data collection was complete, at which time the experimenter was unblinded. Further details of sample sizes/replicates are given in the figure legends. For tamoxifen protocol refinement studies 3 male mice were used per group.

### Targeting constructs

Genomic fragments of the mouse dystrophin gene were obtained from BAC clones, which were isolated by PCR screening of CITB Mouse BAC DNA pool (#96021RG, Research Genetics). As a targeting construct (named E2TV) for introducing a loxP sequence in front of exon 2, a 2.2-kb EcoR I—Afl II fragment for a 5′-homologous arm and a 3.2-kb Afl II—EcoR I fragment for a 3′-homologous arm, both derived from a region ranging from intron 1 (Purkinje) to intron 2, were assembled into a pBluescript IISK(–) plasmid (Stratagene) with three other components: a hygromycin cassette from a pIND/Hygro plasmid (Invitrogen) that is flanked by two FRT sequences; a pgk-thymidine kinase cassette; one loxP sequence. In order to introduce another loxP on a 3′-side of the 3′-untranslated region, the 2nd targeting vector, called E79TV, was constructed by assembling a 4.4-kb Spe I—EcoR I—Bcl I fragment for a 5′-homologous arm and a 1.7-kb Bcl I—EcoR I fragment for a 3′-homologous arm. Both arms were derived from a region ranging from intron 78 to a 3′-side of the 3′-untranslated region via the protocol used forE2TV, except that it contained a neomycin cassette from a pcDNA3 (Stratagene) plasmid flanked by two FRT sequences instead of the flrted hygromycin cassette. Suitable restriction sites for Southern blotting were also introduced at the junction of these fragments.

### Generation of gene-targeted mice

The Not I-linealized E79TV targeting construct was electroporated into W4/129S6 ES cells (Taconic Farms). Successful homozygous recombination over the 5′-side of the insertion was confirmed by Southern blotting. That over the 3′-side was confirmed by PCR with the following primer pair: F1, 5′-GCGGCCGGAGAACCTGCGTGCAATCCATCT-3′ (from a neomycin coding region) and R1, 5′-TTTGTCACCACCGCGACTGGGGAAACTACA-3′ (from the far 3′-side of poly-A signal of dystrophin). The BamH I-linealized E2TV construct was subsequently electroporated into these targeted cells, and successful homologous recombination was confirmed by PCR over each homologous arm with the following primer pairs: F2, 5′ TAGTATTCCCGCATAGACAGGGCACAAATG-3′ (from an intron 1-Purkinje) and R2, 5′-GGAGGGCGTGGATATGTCCTGCGGGTAAAT-3′ (from a hygromycin coding region) over the 5′-homologous arm; F3, 5′-GGCCGATGCAAAGTGCCGATAAACATAACG-3′ (from a hygromycin coding region) and R3, 5′-GCTGGGTTCTGCCCCCTAAACTCCATCATC-3′ (from an intron 2) over the 3′-homologous arm.The presence of coexisting random integrations of the targeting vector in recombinants was ruled out by Southern blot analysis after each targeting step, with a neomycin- and a hygromycin-coding sequence probes detecting only a single band in both of two independent restriction enzyme digests (Sac I as well as Bgl II for the neomycin probe; Hind III as well as Stu I for the hygromycin probe: data now shown).

Transfection of pCAGGS-FLPe (Open Biosystems) successfully deleted the flrted neomycin cassette. The whole dystrophin-floxed ES cell clone (with retention of the hygromycin cassette) was microinjected into C57BL/6J blastocysts, and chimeric mice were obtained. Breeding of the male chimeric mouse with C57BL/6J females produced germline-transmitted heterozygous females of F1 generation, and additional breeding of these F1 females with C57BL/6J males produced F2 hemizygous males and heterozygous females. Backcrossing with C57BL/6J was continued to reach 5 times in total, and the colony was maintained by breeding between hemizygous males and homozygous females under this genetic background.

### PCR genotyping of wild-type, floxed- and deleted-dystrophin mice

Genotyping of each locus was performed by PCR under standard conditions, with the following primers: F4: 5′-ATGATCATGGCAGGAAGCATAGCAGTGTGC-3′; F5: 5′-TTTGGGGTGGAGGCTGTCACTGTGTTACAT-3′; R4 5′-GACCCTCCCGCCTTTGTCTTTTGAGTGTCG-3'.

The F4 and R4 primer pair yielded bands of 358 bp in the dystrophin-deleted locus, while F5 and R4 primer pair produced bands of 604 bp and 451 bp in the floxed-dystrophin and the wild-type loci, respectively.

### Animals

Control (C57BL/6) and *mdx* (C57BL/10 ScSn-*Dmd*^*mdx*^*) mice were obtained from Jackson laboratories (Bar Harbor, ME, USA).*

All mice were housed under the same controlled conditions (12:12 h light–dark cycle) with free access to food and water. All animals were maintained on a C57BL/6 background. Genotyping of TgCre mice was done through Transnetyx service. Male mice were used between 6 and 10 months at the time of study. All of the procedures utilized in this study were approved by the University of Minnesota Animal care and Use Committee.

### Cre-mediated transgene excision

Tamoxifen (Sigma T-5648) was administered by IP injection into TgCre and NTgCre mice. Initially, four distinct protocols were tested: (1) 1 single dose of tamoxifen (40 mg/kg); (2) 5 consecutive doses of tamoxifen (40 mg/kg); (3) 5 consecutive doses of tamoxifen (80 mg/kg); (4) 5 consecutive doses of tamoxifen (160 mg/kg). In preliminary experiments, we determined that 5 consecutive doses of tamoxifen (160 mg/kg) have shown the best efficiency in promoting dystrophin gene excision and was the chosen protocol for the study. Tamoxifen was dissolved in sterile peanut oil to a final concentration of 20 mg/ml.

### Quantitative reverse transcription polymerase chain reaction (RT-qPCR)

For quantitative real time PCR, total RNA was isolated and purified using RNeasy fibrous tissue mini kit (QIAGEM, cat.#74704). The quality and total RNA yield were determined by using a Nanodrop UV–Vis Spectrophotometers and only samples that had an A260/A280 ratio between 2.0 and 2.1 were selected for the cDNA synthesis (using 1 µg total RNA) using SuperScript VILO cDNA synthesis kit (Thermo Fisher Scientific, cat#11754-050). All RT-qPCR were performed using SYBR green PCR master mix kit (Thermo Fisher Scientific, cat# A25742) on a Mastercycle epgradient realplex^2^ machine. Samples were run in biological triplicate. For normalization, *GAPDH* gene expression was used. Quantification of mRNA expression was performed with ΔΔCT method. Primers are as follows: for dystrophin mRNA transcripts: forward: 5′—TGG AGT CTC AGT TAC ATA GAC TGA GAC—3′ and reverse: 5′—GGC TGA CTG CTA TCT GAC CTC TGC—3′. For GAPDH transcripts: forward: 5′—CGT CCC GTA GAC AAA ATG GT—3′ and reverse: 5′—TTG ATG GCA ACA ATC TCC AC—3’.

### Total protein extraction

Total cellular protein was extracted using a method whereby hearts and skeletal muscle tissues were frozen in liquid nitrogen and then pulverized and re-suspended in a lysis buffer containing 1% SDS, 5 mM EGTA and protease inhibitors. Samples were kept on ice, vortexed and centrifuged at 14,000*g* for 5 min and the supernatant was collected. Protein concentration was determined using a BCA assay kit (Thermo Scientific, cat#23227) using BSA (Bovine Serum Albumine) as standard.

### Western blotting

The protein samples were combined with the 4× Laemmli sample buffer and heated (95 °C, 5 min). Sixty micrograms of protein was loaded per sample in a 4–20% Tris–glycine gels for SDS–polyacrylamide gel electrophoresis (Criterion TGX pre-casted gradient gel, Bio-Rad). Protein was then transferred to polyvinylidene difluoride membranes. Membranes were blocked for 1 h with 5% nonfat dry milk in Tris buffered saline and incubated with primary antibodies overnight at 4 °C. The following primary antibodies were used: mouse anti-dystrophin (mid-rod: Millipore, mab1692, 1:200) and rabbit anti-GAPDH (abcam, 0.5 µg/ml). The membranes were then incubated with secondary antibodies (1:5,000 goat anti-rabbit [cat#925-32211] or goat anti-mouse [cat#926-68070] IgG HRP conjugates for 1 h at room temperature and the detection was performed using an Odyssey infrared scanner (LI-COR).

### Echocardiography

Echocardiography was performed without knowledge of mouse status. Mice were initially induced with 2.5–3% isoflurane then maintained anesthetized under 0.5–1.5% isoflurane. Mice were placed on a heated platform and had ECG monitoring during the studies. Parasternal long axis (B-Mode) and mid-papillary short axis (M-mode) views of the left ventricle were acquired using the Vevo2100 ultrasound machine (VisualSonics) and MS550D ultrasound probe (VisualSonics). Measurements were made from mid-papillary short axis views of the left ventricle M-mode images. Ejection fraction and fractional shortening values were calculated through the system software.

### Histopathology

Freshly excised hearts and skeletal muscles were cut in half along the transverse plane and placed into OCT medium to be frozen in liquid nitrogen-cooled isopentane (− 160 °C) and stored at − 80 °C. Short-axis cross-sections of the hearts were sectioned at 7 µm and stained with hematoxylin and eosin using standard histochemical procedures. Images were collected on a Nikon E200 microscope.

### Statistics

All statistical analyses were performed using Prism 5.0c (GraphPad, La Jolla, CA, USA). Comparisons between two groups were made using Student’s two tailed *t *test. When more than one independent variable was tested, two-way ANOVA with a Bonferroni posttest was used to compare groups. Data are shown as mean ± SD.

## Supplementary information

Supplementary information
