# Supplementary material for: Investigations of an inducible intact dystrophin gene excision system in cardiac and skeletal muscle in vivo
Source: Sci Rep. 2020 Jul 3;10:10967. doi: 10.1038/s41598-020-67372-0 (PMC7335168; doi:10.1038/s41598-020-67372-0)
Supplement: Supplementary file 1 — Supplementary information [file 41598_2020_67372_MOESM1_ESM.docx]

**Supporting information**

**Investigations of an inducible intact dystrophin gene excision system in cardiac and skeletal muscle in vivo**

**Addeli Bez Batti Angulski^1^, John Bauer^1^, Houda Cohen^1^, Kazuhiro Kobuke^2^, Kevin P. Campbell^2^, Joseph M. Metzger^1^***

**E-mail:** [metzgerj@umn.edu](mailto:metzgerj@umn.edu)

**Supplementary Figure S1:** Blots of the cropped image shown in **Figure 5.** The cropped areas are labeled with red-dotted boxes. These images represent the grouping of two gels/blots cropped from different parts of the same gel.

**Supplementary Figure S2:** Blots of the cropped image shown in **Figure 6a**. The cropped areas are labeled with red-dotted boxes. These images represent the grouping of two gels/blots cropped from different parts of the same gel.

**Supplementary Figure S3:** Blots of the cropped image shown in **Figure 6c.** The cropped areas are labeled with red-dotted boxes. These images represent the grouping of two gels/blots cropped from different parts of the same gel.

**Supplementary Figure S4:** Blots of the cropped image shown in **Figure 6e.** The cropped areas are labeled with red-dotted boxes. These images represent the grouping of two gels/blots cropped from different parts of the same gel.

**Supplementary Figure S5:** Representative sections of hematoxylin and eosin (H&E) staining of heart and quadriceps after tamoxifen treatment and gene excision. (a) Histopathology of heart tissues after 90 days of tamoxifen (TAM) treatment. (b) Histopathology of heart tissues after 180 days of TAM treatment. (c) Histopathology of quadriceps tissues after 90 days of TAM treatment. TgCre = tamoxifen-treated floxed dystrophin with Cre transgene; NTgCre: tamoxifen-treated floxed dystrophin without Cre transgene.

**Supplementary Figure S6:** Echocardiographic evaluation of left ventricular ejection fraction. (a) Echocardiographic evaluation of left ventricular ejection fraction in floxdDys x αMHC.MerCreMer mice 5, 90 and 180 days after last tamoxifen injection (160 mg/kg on five consecutive days). (b) Echocardiographic evaluation of left ventricular ejection fraction in floxdDys x HSA.MerCreMer mice 5 and 90 days after last tamoxifen treatment. Data are represented as mean ± standard error of the mean. (**P* < 0.05, ***P* < 0.01, ****P* < 0.001). WT: non-treated wild type (C57BL/6J), NTgCre: tamoxifen-treated floxed dystrophin without Cre transgene, TgCre: tamoxifen-treated floxed dystrophin with Cre transgene.


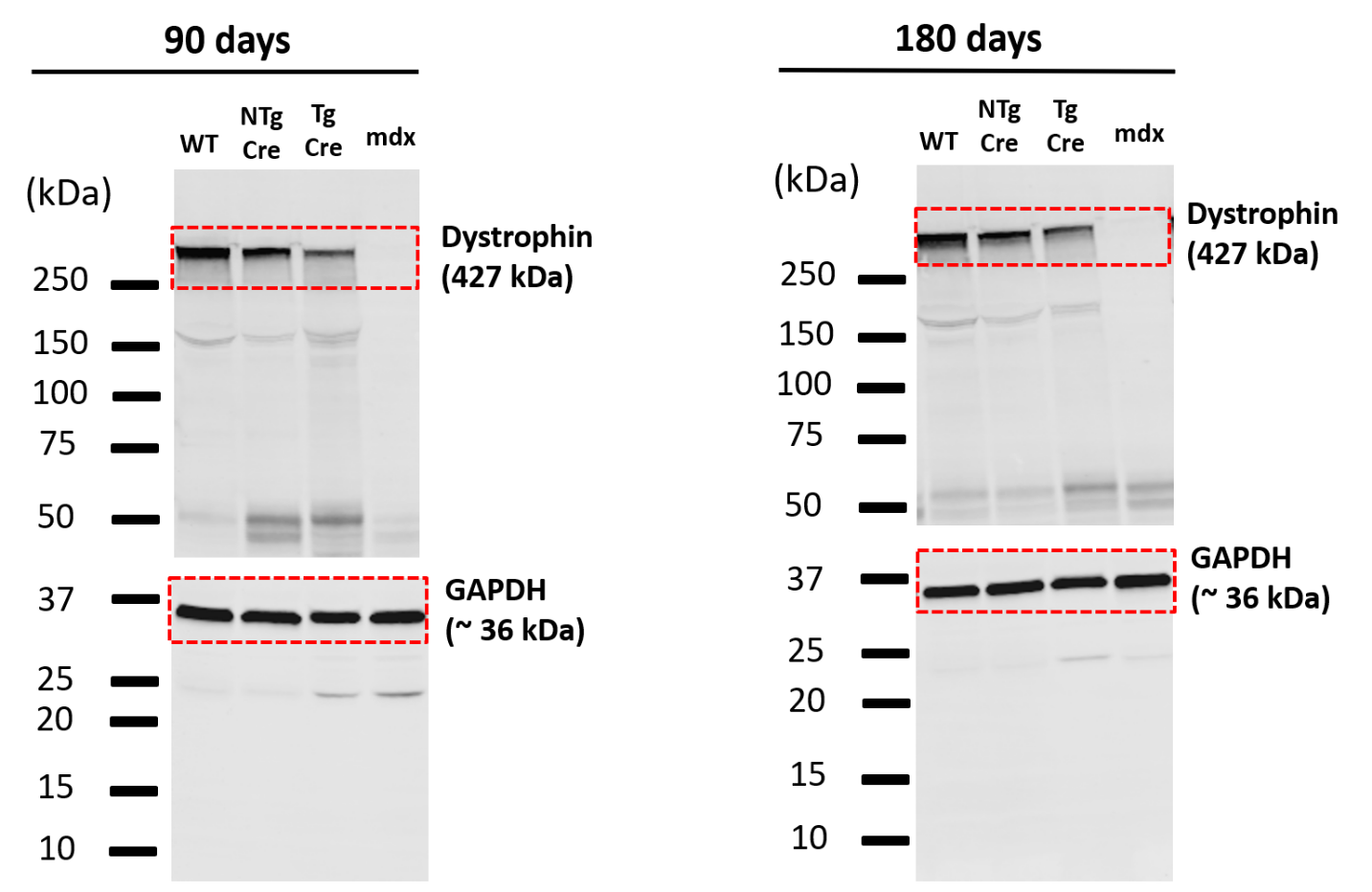


**Supplementary Figure S1:** Blots of the cropped image shown in **Figure 5.** The cropped areas are labeled with red-dotted boxes. These images represent the grouping of two gels/blots cropped from different parts of the same gel.


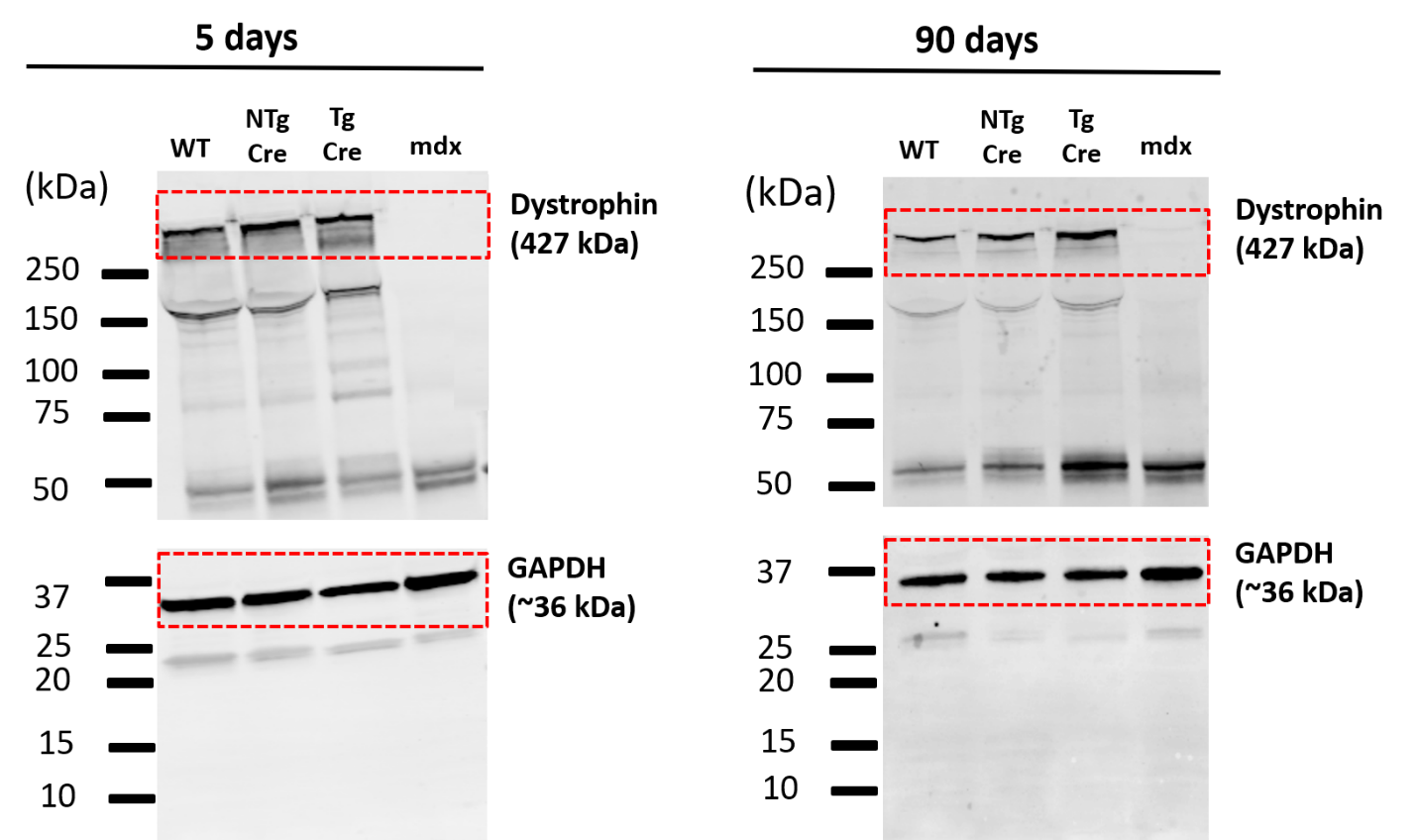


**Supplementary Figure S2:** Blots of the cropped image shown in **Figure 6a**. The cropped areas are labeled with red-dotted boxes. These images represent the grouping of two gels/blots cropped from different parts of the same gel.


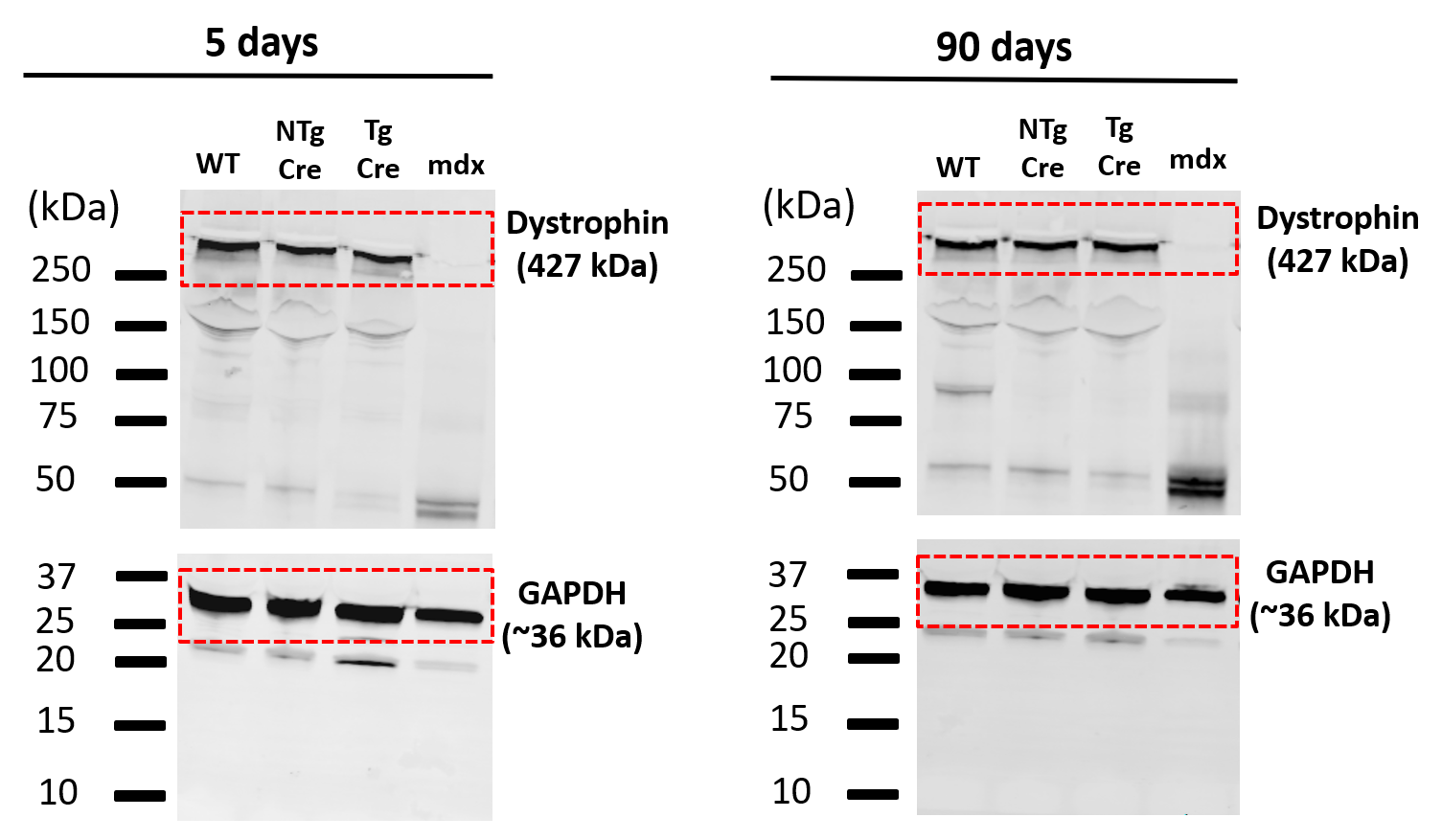


**Supplementary Figure S3:** Blots of the cropped image shown in **Figure 6c.** The cropped areas are labeled with red-dotted boxes. These images represent the grouping of two gels/blots cropped from different parts of the same gel.


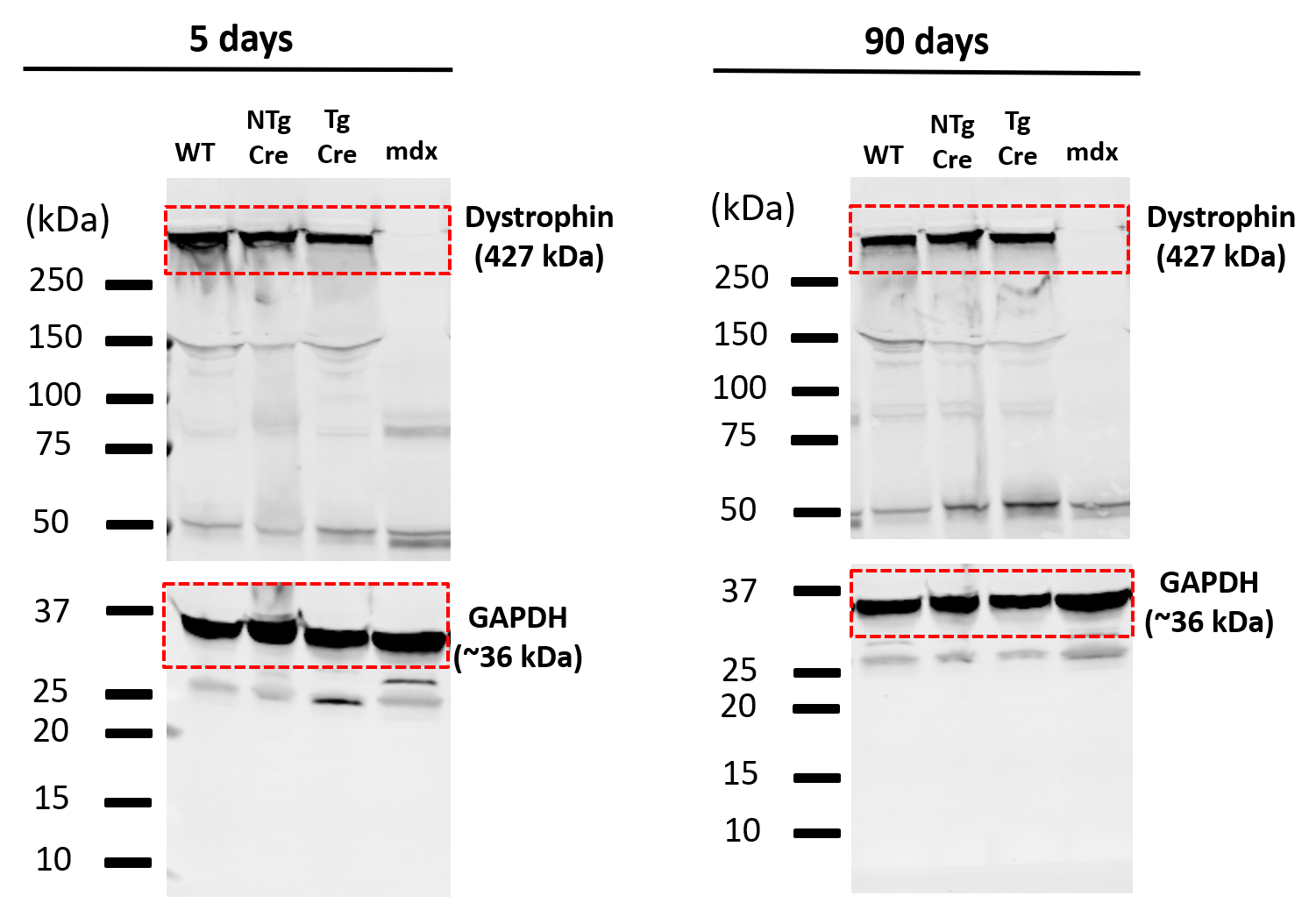


**Supplementary Figure S4:** Blots of the cropped image shown in **Figure 6e.** The cropped areas are labeled with red-dotted boxes. These images represent the grouping of two gels/blots cropped from different parts of the same gel.


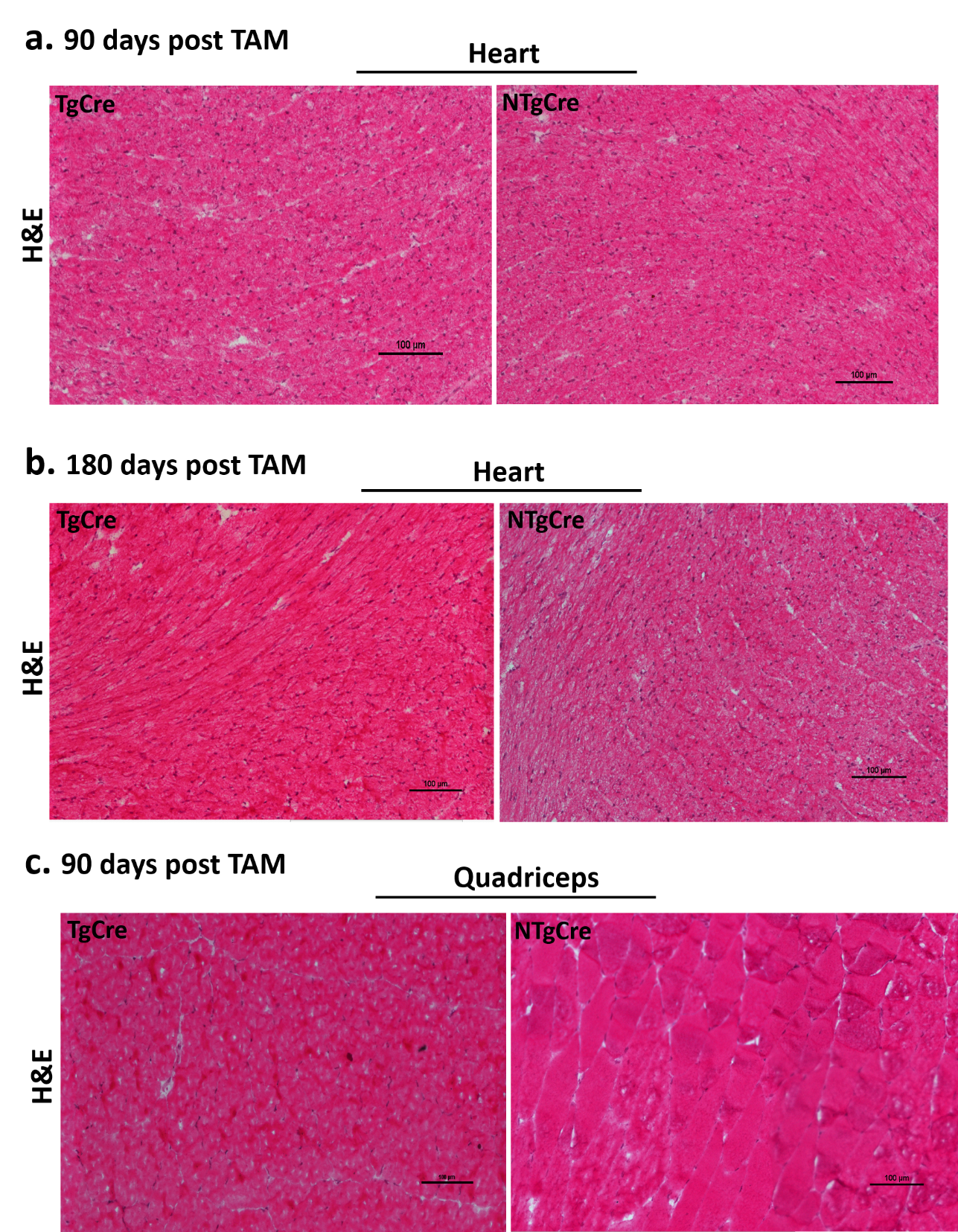


**Supplementary Figure S5:** Representative sections of hematoxylin and eosin (H&E) staining of heart and quadriceps after tamoxifen treatment and gene excision. (A) Histopathology of heart tissues after 90 days of tamoxifen (TAM) treatment. (B) Histopathology of heart tissues after 180 days of TAM treatment. (C) Histopathology of quadriceps tissues after 90 days of TAM treatment. TgCre = tamoxifen-treated floxed dystrophin with Cre transgene; NTgCre: tamoxifen-treated floxed dystrophin without Cre transgene.


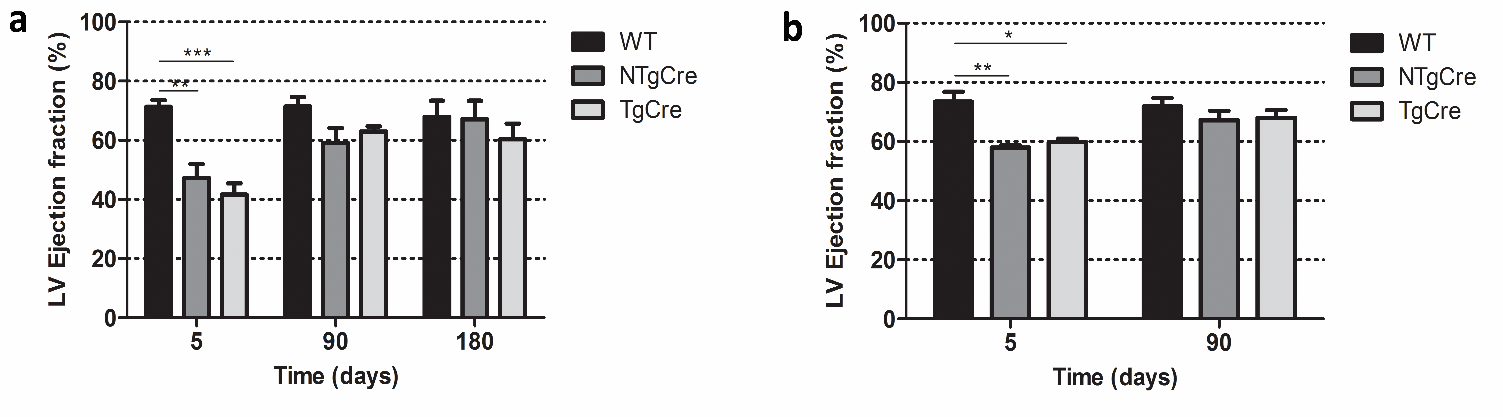


**Supplementary Figure S6: Echocardiographic evaluation of left ventricular ejection fraction.** (a) Echocardiographic evaluation of left ventricular ejection fraction in floxdDys x αMHC.MerCreMer mice 5, 90 and 180 days after last tamoxifen injection (160 mg/kg on five consecutive days). (b) Echocardiographic evaluation of left ventricular ejection fraction in floxdDys x HSA.MerCreMer mice 5 and 90 days after last tamoxifen treatment. Data are represented as mean ± standard error of the mean. (**P* < 0.05, ***P* < 0.01, ****P* < 0.001). WT: non-treated wild type (C57BL/6J), NTgCre: tamoxifen-treated floxed dystrophin without Cre transgene, TgCre: tamoxifen-treated floxed dystrophin with Cre transgene.
